# Fighting Thyroid Cancer with Microgravity Research

**DOI:** 10.3390/ijms20102553

**Published:** 2019-05-24

**Authors:** Marcus Krüger, Daniela Melnik, Sascha Kopp, Christoph Buken, Jayashree Sahana, Johann Bauer, Markus Wehland, Ruth Hemmersbach, Thomas J. Corydon, Manfred Infanger, Daniela Grimm

**Affiliations:** 1 Clinic for Plastic, Aesthetic and Hand Surgery, Otto von Guericke University, 39120 Magdeburg, Germany; marcus.krueger@med.ovgu.de (M.K.); daniela.melnik@med.ovgu.de (D.M.); sascha.kopp@med.ovgu.de (S.K.); christoph.buken@gmx.de (C.B.); markus.wehland@med.ovgu.de (M.W.); manfred.infanger@med.ovgu.de (M.I.); 2 Department of Biomedicine, Aarhus University, 8000 Aarhus C, Denmark; jaysaha@biomed.au.dk (J.S.); corydon@biomed.au.dk (T.J.C.); 3 Max Planck Institute of Biochemistry, 82152 Martinsried, Germany; jbauer@biochem.mpg.de; 4 Institute of Aerospace Medicine, Gravitational Biology, German Aerospace Center (DLR), Linder Höhe, 51147 Cologne, Germany; ruth.hemmersbach@dlr.de; 5 Department of Ophthalmology, Aarhus University Hospital, 8200 Aarhus N, Denmark; 6 Gravitational Biology and Translational Regenerative Medicine, Faculty of Medicine and Mechanical Engineering, Otto von Guericke University, 39120 Magdeburg, Germany

**Keywords:** three-dimensional growth, spheroids, aggressiveness, metastasis, signal transduction, cytokines, target

## Abstract

Microgravity in space or simulated by special ground-based devices provides an unusual but unique environment to study and influence tumour cell processes. By investigating thyroid cancer cells in microgravity for nearly 20 years, researchers got insights into tumour biology that had not been possible under normal laboratory conditions: adherently growing cancer cells detach from their surface and form three-dimensional structures. The cells included in these multicellular spheroids (MCS) were not only altered but behave also differently to those grown in flat sheets in normal gravity, more closely mimicking the conditions in the human body. Therefore, MCS became an invaluable model for studying metastasis and developing new cancer treatment strategies via drug targeting. Microgravity intervenes deeply in processes such as apoptosis and in structural changes involving the cytoskeleton and the extracellular matrix, which influence cell growth. Most interestingly, follicular thyroid cancer cells grown under microgravity conditions were shifted towards a less-malignant phenotype. Results from microgravity research can be used to rethink conventional cancer research and may help to pinpoint the cellular changes that cause cancer. This in turn could lead to novel therapies that will enhance the quality of life for patients or potentially develop new preventive countermeasures.

## 1. Introduction

For cancer biologists, microgravity-based research is still an unusual terrain. Microgravity (µ*g*) provides a physical condition that is not possible on Earth and interestingly, this condition may be ripe for studying cancer [1,2]. The use of µ*g* for cancer research was born at the end of the last millennium. During the STS-90 (Space Transportation System) mission in 1998, primary cultures of human renal cortical cells were cultured for six days aboard the space shuttle Columbia before they returned to Earth for analysis. Hammond et al. [3] reported an alteration of 1632 of the 10,000 analyzed genes relative to ground controls. This was the first experiment to show that reduced gravity can affect a wide range of genes of in vitro cultured cells. These findings led to the speculation that weightlessness could also trigger cancer cells to change the expression of numerous proteins, which could be the basis for the development of new targets for drugs.

Inside the human body, the cells normally grow surrounded by a structure-supporting extracellular matrix together with a regulating biochemical microenvironment, which allows organs, as well as tumours, to maintain their three-dimensional (3D) shapes. Under normal laboratory conditions, adherent cells in vitro do not behave similarly to how they would react in vivo in the body: They grow two-dimensionally (2D), spreading out into monolayers on Petri dishes or polystyrene surfaces, which poses problems for scientists who study cancer by examining genetic changes affecting cell growth and development [4]. During the last decades, scientists have developed several laboratory methods to mimic the 3D growth of cancer cells.

Scaffold-based, spinner flask, liquid-overlay and hanging drop techniques were used to gain multicellular spheroids (MCS). But from a certain size, these MCS showed necrosis inside [5]. Under µ*g* conditions, cells can arrange themselves scaffold-free into large MCS without any signs of necrosis [6]. MCS can help to develop new cancer treatment strategies, which might be later translated into in vivo models. Today they represent a useful model for studying angiogenesis mechanisms and performing pharmacological testing of chemotherapeutic agents such as tyrosine kinase inhibitors (lenvatinib, sunitinib, sorafenib etc.), which are often used in metastatic cancer therapy [7]. In addition, they can be applied in toxicological and radiation experiments [8,9].

**Figure 1:**
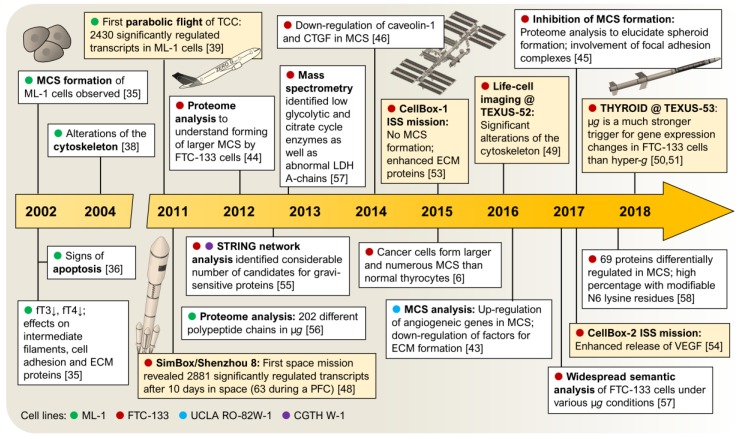
Timeline: research on thyroid cancer in microgravity. White squares: studies in simulated µ*g*; yellow squares: studies in real µ*g*. PFC: parabolic flight campaign, TCC: thyroid cancer cells.

Thyroid cancer is the most common form of endocrine malignancy. Over the past four decades, incidence rates have increased worldwide [10]. According to the Global Cancer Statistics GLOBOCAN, 567,233 new cases were diagnosed in the world population, and 41,071 people died from thyroid cancer in 2018 [11]. Poorly differentiated thyroid tumours are aggressive and metastasize early, resulting in poor prognosis. Also, differentiated (papillary or follicular) thyroid cancer, that is usually well treatable, could—in its recurrent form—become less-differentiated by diminishing its iodine uptake capability. Thus, current treatment options for recurrent differentiated thyroid cancer are extremely limited and patients show remarkably reduced survival. Scientists have searched intensely for new ways/methods to identify targets for novel drugs—and some of them have considered to use experimentation in altered gravity conditions (Figure 1) [12,13].

## 2. Ground-Based Techniques Allow Microgravity Research on Earth

**Figure 2:**
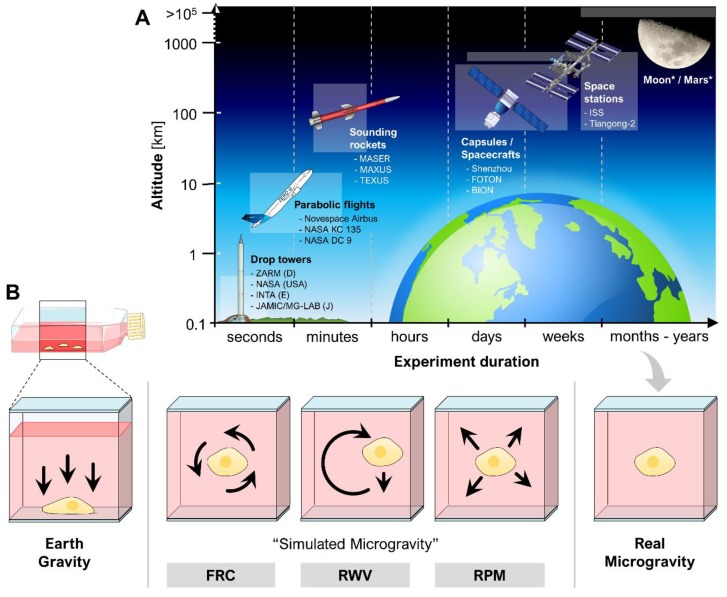
(**A**) Currently available and future (*) platforms for research in real µ*g*. The highlighted boxes indicate altitude and duration ranges of the experiments; (**B**) Different ground-based facilities to simulate µ*g* on Earth. Arrows indicate gravitational forces (Earth gravity) or movements to annulling gravity (“simulated µ*g*”). Parts of the figure were drawn by using pictures from Servier Medical Art, licensed under a Creative Commons Attribution 3.0 Unported License (https://creativecommons.org/licenses/by/3.0/).

Experiments in real µ*g* (parabolic flights, sounding rocket flights, experiments on satellites, space vehicles or space stations (Figure 2A)) are rare and expensive. For this reason, different ground-based facilities have been developed to simulate µ*g* on Earth [1]. Devices such as the fast-rotating clinostat (FRC) [14], the rotating wall vessel (RWV) [15] or the random positioning machine (RPM) [16,17] allow cost-efficient preparations of spaceflights as well as intensive research in stand-alone studies (Figure 2B). The ground-based facilities have been shown to imitate µ*g* effects for several (but for not all) experimental conditions [18,19].

## 3. The Behaviour of Normal Thyrocytes in Microgravity

The structural feature of the thyroid tissue is the thyroid follicle. These follicles are formed by the cells that produce the thyroid hormones triiodothyronine (T3) and thyroxine (T4) and are called follicular epithelial cells or thyrocytes. In vivo, the thyrocytes are arranged in a single layer (epithelium) and surround the lumen of the follicles. In cross sections, the follicles of the healthy thyroid gland are round or oval. Microgravity changes the thyroid follicles [20]. The follicles reveal larger thyrocytes and show an elevation of cyclic adenosine monophosphate (cAMP), thyrotropin-receptors and caveolin-1 [20,21]. Earlier in vitro and in vivo experiments demonstrated that thyrocytes respond to µ*g* and hypergravity conditions [22,23,24,25,26]. Kossmehl et al. [23] showed that normal HTU-5 cells (obtained without transfection) [27] exposed to clinorotation revealed signs of apoptosis, as demonstrated by activation of caspase-3, increases in Fas and Bax and elevation of 85-kDa apoptosis-related cleavage fragments resulting from enhanced poly(ADP-ribose) polymerase activity. Apoptosis was found for various different cell types exposed to altered gravity conditions [28,29,30,31]. Results of the TEXUS (German: ‘Technologische EXperimente Unter Schwerelosigkeit’)-44 sounding rocket mission with FRTL-5 cells onboard showed that in real µ*g*, normal thyroid cells do not respond to thyroid-stimulating hormone (TSH) treatment and present an irregular shape with condensed chromatin, a modification of the cell membrane with shedding of the TSH receptor in the supernatant, as well as elevated sphingomyelin-synthase and Bax protein [22]. With the help of a rotary cell culture system, differentiated thyroid neofollicles were located in close proximity after two weeks of culture [24]. A time-dependent increase in human thyroglobulin was detected in the cell culture supernatant. Exposure of thyrocytes in a rotary cell culture system under the presence of keratinocyte growth factor (KGF) allowed the formation of artificial human thyroid organoids. They structurally resembled natural thyroid tissue [24]. In addition, Nthy-ori 3-1 thyrocytes were able to form 3D spheroids when exposed to an FRC or an RPM [25]. This cell line was originally obtained from normal human primary thyrocytes transfected with a plasmid containing an origin-defective large T antigen SV40 genome [32]. Although Nthy-ori 3-1 cells remain non-tumorigenic, they possess a few specific features such as a strongly diminished thyroglobulin production and the presence of tri-tetraploid clones. Nthy-ori 3-1 cells were exposed for 24 h and 72 h to an RPM. After a 24-h RPM-exposure, they started to detach and to form 3D aggregates. The spheroids increased in number and size over time (72 h), while adherent cells decreased [26]. Nthy-ori 3-1 cells secreted various cytokines in connection with focal adhesion proteins and revealed positively or negatively interacting proteins, strengthening the onset of MCS formation. The interleukins (IL)-6, IL-8 and TIMP-1 seemed to be involved in this process [26]. Long-term RPM-exposure of Nthy-ori 3-1 cells demonstrated no signs of necrosis in the centre of the spheroids [6]. The non-malignant thyroid cells did not express receptors for vascular endothelial growth factor (VEGFRs) in a measurable amount [6] but they reacted with an up-regulation of downstream signalling molecules, which hinted either to a delayed reaction to the simulated µ*g* or to further signalling molecules, which are involved in 3D-aggregate formation. The multipotent IL-6 could be a candidate that influences the cytoskeleton via β-actin [6]. IL-6 is known to be involved in angiogenesis and metastasis not only in thyroid cancer but also in several other cancer types. Elevated IL-6 concentrations could be explained by mechanically triggered activation of the specific surface receptor CD44 [33]. It was suggested that the CD44-IL-6-connection plays a possible role in gravity perception [6]. At least in pancreatic cancer cells, IL-6 stimulation activates the small GTPase cell division cycle 42 (Cdc42) and thus promotes the formation of pre-migratory, actin-based filopodia [34].

In summary, normal thyrocytes revealed changes in the cytoskeleton, shredding of their membranes, alterations in gene expression, protein synthesis and secretion and showed an increased amount of apoptosis and a change in growth behaviour.

## 4. Research on Thyroid Cancer Cells in Microgravity

**Figure 3:**
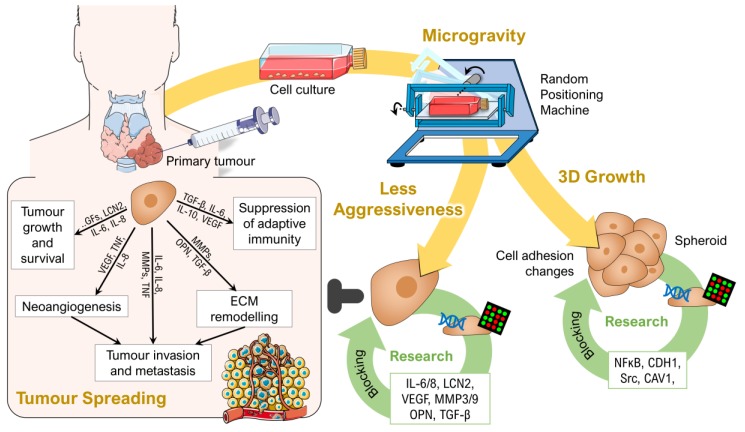
Overview: Current research on thyroid cancer using (simulated) microgravity. Parts of the figure were drawn by using pictures from Servier Medical Art, licensed under a Creative Commons Attribution 3.0 Unported License (https://creativecommons.org/licenses/by/3.0/).

Research on thyroid cancer cells using µ*g* conditions became a more and more interesting topic [1]. Nearly 20 years of µ*g* research in this field provided a deeper insight into the growth behaviour and signalling mechanisms of thyroid cancer (Figure 3). Since the first experiments in 2002, many studies have been conducted both in real and simulated µ*g* environments using different model cell lines (ML-1, FTC-133 and UCLA RO-82W-1) and different research platforms (see also Figure 1).

- Ground-Based Studies

**Figure 4:**
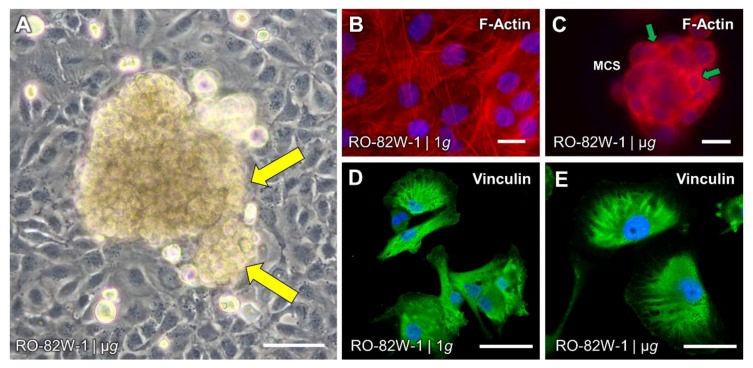
Simulated microgravity affects morphology and growth of thyroid cancer cells: (**A**) Multicellular spheroid (MCS) of UCLA RO-82W-1 cells, observed after three days on a random positioning machine (RPM). Yellow arrows indicate proliferating areas; (**B**,**C**) Fluorescence staining of F-actin (red) in UCLA RO-82W-1 cells that were grown for three days in normal gravity (**B**) or on an RPM (**C**). Small MCS were visible with increased F-actin deposits at the outer membranes of the MCS surface (green arrows); (**D**,**E**) Immunofluorescence of vinculin (green) in UCLA RO-82W-1 cells. DAPI-stained nuclei are shown in blue. Scale bars: 50 µm.

Growth of 3D-aggregates (Figure 3 and Figure 4A) during random positioning was observed very early [35] and it was found that apoptosis is involved in MCS formation [23,36]. Programmed cell death of µ*g*-exposed cells was also confirmed for other cell types [30,37]. In this setting, nuclear factor kappa-light-chain-enhancer of activated B cells (NFκB) was identified as a key player [37]. Furthermore, simulated µ*g* affected gene expression patterns and the structure of the cytoskeleton already very early after exposure to µ*g* [38,39]. In addition to alterations of the F-actin morphology (Figure 4B,C), 30 min of clinorotation induced a disorganization of vimentin, cytokeratin and microtubules. However, these changes were nearly reversed after 48 h [40]. This finding clearly supported the hypothesis that the cytoskeleton may sense gravity in eukaryotic cells [41]. µ*g* also increased the amount of extracellular matrix (ECM) proteins in a time-dependent manner [40]. Based on the proteome data of different µ*g* experiments, Bauer et al. [42] postulated that the stability of the ECM has a great influence on MCS formation under µ*g*.

For scientists, it is very important to understand why and how MCS were formed. A 24 h-RPM-experiment with UCLA RO-82W-1 cells showed that genes of products involved in angiogenesis were up-regulated in MCS whereas gene products involved in ECM formation were down-regulated [43]. Especially FTC-133 follicular thyroid cancer cells formed larger and numerous spheroids compared to normal thyrocytes [6]. It is assumed that these cells express surface proteins that bind fibronectin, strengthening 3D cell cohesion [44]. An intense proteome analysis of FTC-133 cells to elucidate MCS formation revealed that paxillin, vinculin (Figure 4D,E) and focal adhesion kinase (FAK or PTK2) may be positioned within the focal adhesion complex in a way that favours cell detachment from the bottom of a culture flask and mutual attachment [45]. Caveolin-1 and connective tissue growth factor (CTGF) were down-regulated in spheroids [46].

Different transcription factors, especially the sex-determining region Y-related high-mobility group box (SOX) proteins, are deeply involved in cancer development and metastasis but for thyroid cancer only a few studies have addressed this topic so far. Also, the influence of µ*g* on SOX expression is still unclear. In a first experiment, FTC-133 cells were analyzed after 10 days on the RPM. The cell line did not express *SOX2*, *SOX5*, *SOX6* and *SOX7* but was positive for SOX9 and SOX11. RPM-exposure had only a marginal effect on the expression of the SOX genes both in adherently growing cells and in the MCS cells [47].

- Parabolic and Sounding Rocket Flights

Alternating phases of real µ*g* (ca. 22 s), hypergravity and normal gravity during a parabolic flight provide optimal conditions for investigating morphological and genetic alterations directly in the transition from hypergravity to µ*g*. A parabolic flight changed the morphology of the F-actin/cytokeratin cytoskeleton of ML-1 cells as well as the expression status of genes involved in forming the cytoskeleton and ECM [39]. Interestingly, the gene expression during the parabolic flight (and thus several changes in gravity conditions) was often regulated in the opposite direction compared to the RPM or space [48].

Several sounding-rocket flights allowed further short-term experiments in real µ*g* (about 6 min) during the last years. The focus of these analyses was the very early mechanisms of cells exposed to real µ*g* aiming to detect the first steps that later lead to cell detachment and MCS formation. FTC-133 cells expressing the LifeAct-GFP marker protein for the visualization of F-actin were flown on TEXUS-52 in 2015 and observed with live-cell imaging using the confocal FLUMIAS (Fluorescence-Microscopic Analysis Systems for Space Application) microscope. They proved significant structural alterations of the actin cytoskeleton as well as effects on the gene expression of genes involved in cytoskeleton forming (*EZR*) and signalling (*SEPT11*) [49]. With the *THYROID* project on the TEXUS-53 mission in 2016, Kopp et al. [50] investigated the effects of short-term µ*g* and hypergravity on the transcriptome and proteome of FTC-133 cells. They identified a possible role of epidermal growth factor (EGF) and vascular endothelial growth factor (VEGF) for spheroid formation in µ*g*. In addition, they found that µ*g* is a stronger regulator of gene expression than hypergravity, which shortly occurs during the launch of the rocket [51].

- Space Missions

The long-term experiments in real µ*g* provided a deeper insight into the growth behaviour of thyroid cancer cells in weightlessness excluding any additional shear forces as they might be induced in ground-based facilities. This helped to evaluate whether µ*g* was the trigger for the effects observed in RPM experiments on Earth. After the SimBox/Shenzhou-8 mission in 2011, it became clear that space-flown follicular thyroid cancer cells changed their proliferation, cell adhesion, growth behaviour, and the post-flight results indicated that the cells shifted toward a less-aggressive phenotype [48]. Another interesting finding was that the confluence status of a cell culture could influence MCS formation. Preincubation of FTC-133 cells for five days before exposure to µ*g* during the CellBox-1 mission completely inhibited the spheroid formation suggesting that the cell density together with high amounts of ECM proteins and caveolin-1 are of great importance for 3D growth [52,53]. The CellBox-2 mission in 2017 allowed to investigate the effects of real µ*g* on the transcriptome and proteome of FTC-133 cells. The cells formed MCS in space and interestingly, these spheroids differed from MCS generated on an RPM by an enhanced release of VEGF [54]. As VEGF has a key role in neoangiogenesis, the enhanced production suggests a different angiogenic potential of thyroid cancer cells in space.

- Supporting Semantic Analyses

Genomic and proteomic analyses performed after exposing healthy and malignant thyroid cells to devices simulating µ*g* or to space or orbit flights unveiled a number of genes and proteins, which may play a role when the cells adapt to µ*g* [55,56,57]. Semantic analyses of these genes and proteins enabled the identification of groups of interacting cellular entities and members of distinct pathways [58,59]. A group of gravisensitive genes was detected, which altered their transcription when exposed to µ*g*. They code for proteins, which are either secreted into the culture supernatant, inserted in the cell membrane or located within the cytoplasm. Within the first 24 h of culturing the cells on an RPM, genes coding for secreted proteins were up-regulated but those coding for membrane or cytoplasmic proteins were down-regulated. After prolonged incubation, up-regulation of genes coding for membrane and cytoplasm proteins was also observed. According to information retrievable from the literature, these proteins form a network of interaction. They can either mutually influence their gene expression, form complexes or regulate their activity. Interestingly, this interaction network not only includes the products of gravisensitive genes but it also comprises proteins detected in culture supernatants by multianalyte profiling technology upon the cells’ exposure to µ*g* [6,25,43,52].

Another group of related proteins was found when a deep proteome analysis was evaluated. This analysis was performed comparatively on thyroid FTC-133 cells, which were either incubated under normal gravity conditions or on the RPM, where they were split into two populations, one continuing to grow adherently, while the other one started to grow within a 3D aggregate. The evaluation showed 104 proteins of the integrin signalling pathway. In cells forming 3D aggregates during three days of culture on the RPM, the levels of caveolin-1 and p130cas proteins were reduced but arf-GAP with SH3 domain (ASAP1) production was enhanced. The results confirmed that caveolin-1 plays an important role in spheroid formation [52] and challenged the speculation that the positions of the proteins paxillin, vinculin and focal adhesion kinase may be changed by µ*g* within the focal adhesion complex in a way that favours cell detachment from the bottom of a culture flask. Preliminary hints towards a µ*g*-dependent change of intracellular vinculin distribution had already been observed during a recent TEXUS flight [45,51]. Taking together, in vitro studies on thyroid cells exposed to microgravity suggested that groups of proteins are affected by µ*g* which interact over the membrane barrier and provided first insights in the mechanisms of tissue cells leaving the site of growth to migrate to another one as observed in metastasis [60].

### 4.1. Spheroids as a 3D Tumour Model

**Figure 5:**
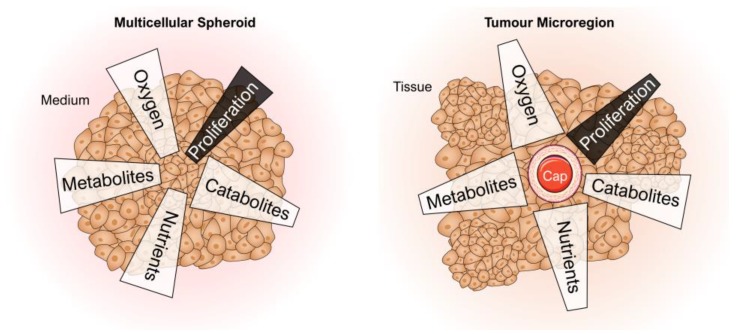
Schematic of the analogy between a multicellular spheroid (**left**) and a tumour (**right**) to illustrate the pathophysiological inside-out situation described by Kunz-Schughart [62]. Cap: capillary. Parts of the figure were drawn by using pictures from Servier Medical Art, licensed under a Creative Commons Attribution 3.0 Unported License (https://creativecommons.org/licenses/by/3.0/).

Both thyroid cancer cells and normal thyrocytes formed MCS [43,61], when they were cultivated in (simulated) µ*g*. Follicular thyroid cancer cells detached from the cell culture flask bottom and assembled to compact 3D aggregates within a short time [35]. The tumour MCS were described to mimic small metastases that are not as complex as the primary tumour but could resemble a part of their organ of origin, reflecting a pathophysiological ‘inside-out’ situation (Figure 5) [62]. The ECM of MCS differed in amount and composition from the corresponding monolayer cultures comprising a complex 3D network of cell-cell and cell-matrix interactions. These differences might facilitate the penetration and action of drugs and affect the distribution and function of other physiological molecules such as hormones and growth factors. Thus, the composition of MCS fundamentally determines the regulation of cell growth, differentiation and apoptosis [62].

An important task is to clarify the mechanisms for spheroid formation. Several factors are involved in this process in addition to their role in metastasis and spreading of cancer cells in vivo (Table 1 and Table 2). Some of these factors may serve as future targets in cancer therapy.

**Figure 6:**
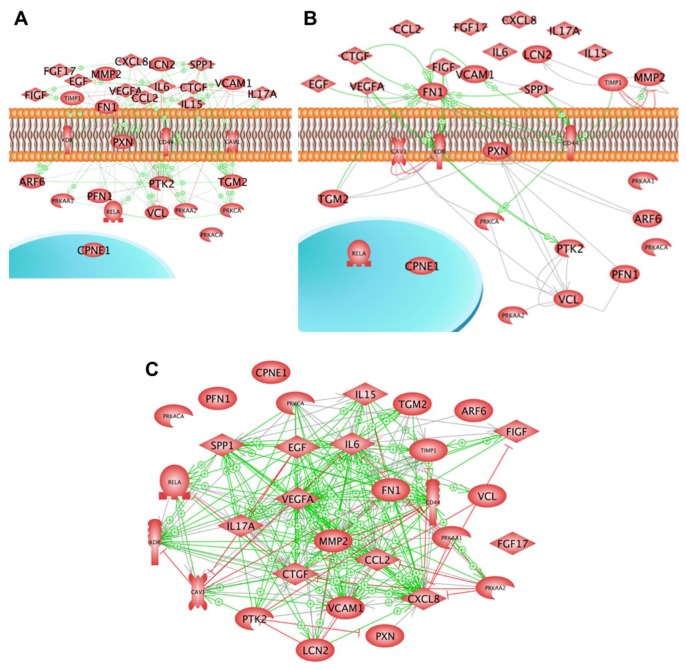
Semantic analysis of factors involved in spheroid formation: (**A**) Protein-protein regulation of follicular thyroid cancer cells exposed to µ*g*; (**B**) Direct protein interactions; (**C**) Gene interaction of various differentially regulated genes of thyroid cancer cells exposed to µ*g* conditions. Connecting lines indicate binding, arrows show directed interaction; green lines with ‘+’ signs point to an enhancing effect, red lines with perpendicular bars indicate inhibition. Networks were built up using Pathway Studio v.11 (Elsevier, Amsterdam, The Netherlands).

An interesting finding was that caveolin-1 is a key factor for the inhibition of spheroid formation when confluent monolayers are exposed to µ*g* [52]. The results of a pathway analysis using the data of these articles (Table 1) are given in Figure 6.

Ten of numerous studies on the behaviour of thyroid cells revealed proteins and genes coding for proteins that appear to be key proteins in spheroid formation. The studies were performed at very different time points on different populations of thyroid cells, culturing the cells either on an RPM, a clinostat or in space. The 31 proteins recognized as being important in MCS formation were, besides copine-1, all members of the networks of interaction according to the literature evaluated by the Elsevier Pathway Studio.

As demonstrated in Figure 6, various factors are involved in the formation of spheroids. The next step would be to study them in more detail. A first step was done by targeting E-cadherin in MCF-7 breast cancer cells [65]. The proto-oncogene tyrosine-protein kinase c-Src was detected in MCF-7 MCS. Anti-E-cadherin antibodies had enhanced the amount of MCS. The agent PP2, blocking the E-cadherin reducer SRC, prevented MCS formation completely [65].

Figure 6A,B show protein interaction networks. Looking at the mutual regulation of the proteins, it can be seen that 29 proteins form a network influencing the activity of each other (see Figure 6A). In most cases, the influence is stimulating (green arrows). Only a few red lines with little perpendicular bars at their ends indicate inhibition. PTK2, the focal adhesion kinase, seems to play a central role in this system, as most of the arrows point to its icon. As the icon of VEGF-A is a starting point for a considerable number of green arrows, one may conclude that VEGF-A is a strong initiator of spheroid formation. Interestingly, 21 of the proteins investigated bind physically to each other forming complexes (connecting lines) or triggering the partner’s activity (green arrows) (Figure 6B). In two cases, TIMP1-MMP2 and CAV1-KDR inhibition were indicated (red lines with the perpendicular bar at the side of inhibited protein). In this system of binding, fibronectin seems to play a central role.

Figure 6C shows that 27 of the proteins recognized as being important in spheroid formation influence each other’s mRNA expression. As shown by the high number of arrows the mutual gene expression regulation is very intensive. Again, most proteins favoured the expression of the partner protein’s mRNA (green arrows). Still a considerable number inhibits (red line with perpendicular bar at the end). In this system *VEGFA*, *KDR*, *IL6* and *CXCL8* are indicated as dominant target genes as many arrows point to their icons. Noteworthy is that many green arrows start at the SPP1 icon. This suggests that osteopontin is a major trigger of spheroid formation.

**Figure 7:**
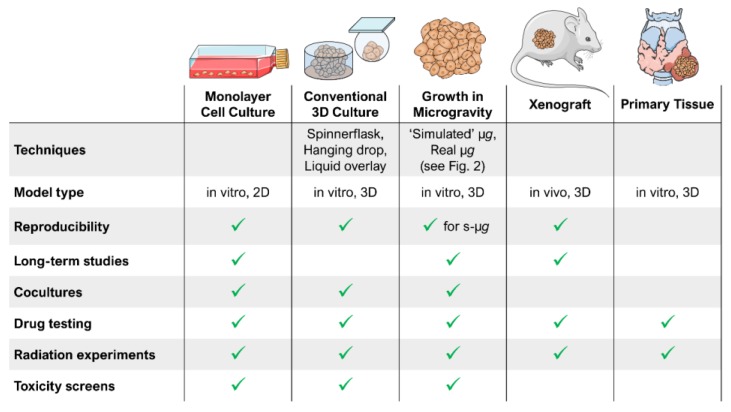
Comparison of different cell culture models for thyroid cancer research. s-µ*g*: simulated microgravity. Parts of the figure were drawn by using pictures from Servier Medical Art, licensed under a Creative Commons Attribution 3.0 Unported License (https://creativecommons.org/licenses/by/3.0/).

Figure 7 provides a comparative overview of the current cell (culture) models used for cancer research. It visualizes possibilities and limits of the different model types including µ*g*-grown tumour spheroids. The µ*g*-based method is able to provide a large number of MCS in a short time and is suitable for long-term experiments (no necrotic cores) as well as for cocultures. MCS were used for a variety of experimental studies using radiotherapy, chemotherapy, radioimmunotherapy, cell- and antibody-based immunotherapy, hyperthermia, gene therapy and photodynamic treatment [66]. Further, they serve as an invaluable experimental and theoretical model for basic research on proliferation, viability, energy metabolism, nutrient metabolism, invasion, cell-cell interactions and extracellular matrix composition helping to better estimate in vivo antitumor treatment modalities [66]. But although thyroid cancer cells grow in 3D structures in µ*g*, MCS do not possess blood vessels that can provide oxygen and nutrients. Therefore, the MCS size remains limited.

### 4.2. Growth of Thyroid Cancer Cells in Microgravity–A Temporary Mimicry of Metastasis?

When a (progressive) tumour spreads from its primary site to another part of the organism this process is called metastasis. Metastatic cancer is characterized by the same type of cancer cells as the primary cancer. The tumour starts to extend and invades into the structures and tissues in its surrounding area. The first description of a thyroid metastasis was made by Rudolf Virchow in 1871, who found metastatic thyroid cells inside a testis [67]. Thyroid cancer mostly spreads to the lymph nodes, lungs, bone and occasionally to the brain. Follicular thyroid cancer tends to spread to distant sites via the blood vessels (hematogenously), as opposed to papillary thyroid cancer, which metastasizes through the lymphatic system (lymphogenously), usually to local lymph nodes. As angioinvasion is associated with the risk of recurrence, the differentiation of lymphatic and angioinvasion is of high importance [68]. Survival and growth of metastases at the new sites needs angiogenesis: The tumour forms a new blood supply by building new blood vessels. Metastasis is responsible for more than 90% of all the cancer-associated deaths. Approximately 30% of patients suffering from thyroid cancer develop metastases [69] and the incidence of distant metastasis after thyroidectomy remains between 7% and 23% [70]. In rare cases metastasis is associated with malignant ascites (especially described for medullary thyroid cancer [71]). Metastatic ascites containing multicellular spheroids can promote chemo-resistance and recurrence [72]. Model mechanisms relating to how single cells aggregate to form MCS once detached are important. MCS have been used in developmental biology and experimental cancer research for many years [73]. They are used as in vitro models of tumour microregions and as early avascular stage of tumour growth [62,66,74].

Progression and aggression of cancer are known to be closely connected to mechanical stress [75] resulting in alterations of cytoskeletal structures, cellular shape, proliferation, differentiation, cell adhesion, survival/death and migration [76,77]. The loss of gravity can also be a stressor for adherently growing cells that may be induced by tensegrity (tensional integrity) after gravitational unloading [78,79]. Interestingly, there are many parallels between the detachment of single cells from the primary tumour during metastasis and the detachment of adherently growing cells in µ*g* (Table 3). Whereas metastasis is forced, among other factors, by the external pressure from the growing tumour [76], a possible physical trigger for cell detachment in µ*g* is the lapse of gravity.

Research using MCS focused on mechanisms involved in proliferation, invasion and metastasis. µ*g* conditions have been shown to induce 3D growth of benign and malignant cells [72]. A great advantage of µ*g*-engineered MCS is that they do not develop a necrotic centre when cultured for several weeks [6,80]. Interestingly, results hint to a re-differentiation of follicular thyroid cancer cells in space to a less-aggressive phenotype (see chapter 4.4.) [48]. These findings are supported by recent data obtained with melanoma cells types exposed to µ*g*. BL6-10 cells did not form multicellular spheroids but revealed comparable results, as clinostat-simulated µ*g* reduced proliferation, adhesion and invasiveness in vitro and decreased tumour lung metastasis in vivo. Clinorotation also down-regulated metastasis-related integrin α6β4, MMP9 and Met72 molecules. Further, it significantly reduced the formation of focal adhesions and activation of the focal adhesion kinase (FAK), Rho family proteins (RhoA, Rac1 and Cdc42) and mTORC1 kinase but activated AMPK and ULK1 kinases [81]. Clinostat-exposure of the cells inhibited focal adhesions and induced inhibition of FAK and RhoA signalling and the mTORC1 pathway, which resulted in the activation of the AMPK pathway and thus reducing melanoma cell proliferation and metastasis. µ*g* further changed the cytoskeleton and nuclear positioning, leading to enhanced cell apoptosis by suppressing the FAK/RhoA-regulated mTORC1/NFκB and ERK1/2 pathways [82]. Similar data were found by Grosse et al. [61] investigating follicular thyroid cancer (FTC-133) cells. Especially invasive follicular thyroid cancer is known to show an extensive vascular invasion [68]. FTC-133 cells grown on the RPM showed higher levels of NFκB p65 protein and apoptosis than controls grown under normal gravity (1*g*), a result also found earlier in endothelial cells [31]. *IL6*, *CXCL8*, *CD44* and *SPP1* were significantly up-regulated in adherent cells but not in MCS, while *ERK1/2*, *CAV2*, *TLN1* and *CTGF* were significantly down-regulated in adherent cells. Simultaneously, the expression of *ERK2*, *IL6*, *CAV2*, *TLN1* and *CTGF* was reduced in 3D MCS compared to 1*g* samples. The signalling elements IL-6, IL-8, OPN, TLN1 and CTGF were involved together with NF-κB p65 in the RPM-dependent thyroid carcinoma cell MCS formation. Similar findings were reported with glioma cells exposed to µ*g* [83]. Simulated µ*g* induced apoptosis of U251 cells. The FAK/RhoA/Rock and FAK/Nek2 signalling events were attenuated by simulated µ*g* to destabilize the actin cytoskeleton and centrosome disjunction, which caused G2/M arrest and inhibition of cell viability and migration [83]. The FAK/RhoA/Rock and FAK/Nek2 pathways are affected by simulated µ*g* and might serve as future targets. In addition, an earlier study showed that simulated µ*g* reduced the metastatic potential of human lung adenocarcinoma cells by diminishing the expression of antigen MKI67 and MMP2, thereby inhibiting cell proliferation, migration and invasion [84]. In addition, MMP2 was reduced in the thyroid cancer space samples [48], supporting the results of Chang et al. [84] that simulated µ*g* exerts an antiproliferative effect on lung cancer cells.

### 4.3. Reduced Aggressiveness of Thyroid Cancer Cells after Long-Term Exposure to Real Microgravity

**Figure 8:**
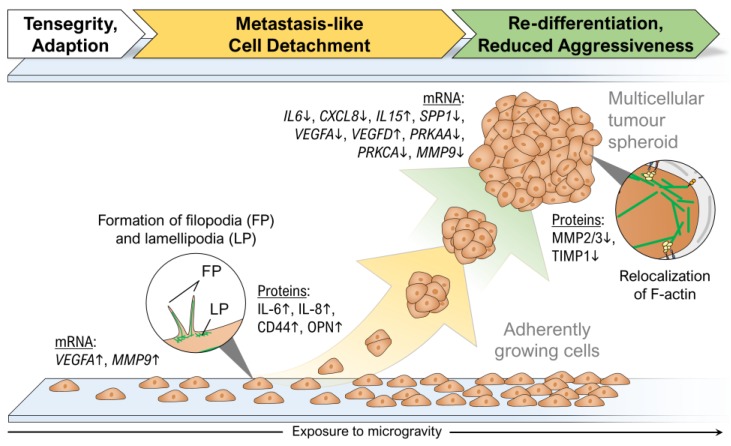
From metastasis-like cell detachment to lower aggressiveness: the transformation of thyroid cancer cells during their exposure to microgravity. The green lines show F-actin filaments. ↓ down-regulation/decrease, ↑ up-regulation/increase.

When thyroid cancer cells had been examined after cultivation on an RPM at various time-points results suggested that µ*g* initiated a re-differentiation of dedifferentiated FTC-133 cells after long-term RPM-exposure (Figure 8) [6]. The exact mechanisms behind this transformation are still unknown. After 7 and 14 days, expression of angiogenic factors like VEGF-A was significantly down-regulated, whereas the expression of apoptotic factors was increased. This suggested that exposure to simulated µ*g* enabled selective programmed cell death, which identified the cells more differentiated compared to those grown under normal conditions [6]. Similar results were obtained from the SimBox/Shenzhou-8 mission when FTC-133 cells were cultured on an unmanned spacecraft for two weeks. Space-flown cells were shifted towards a less-malignant phenotype by down-regulation of *VEGFA* and up-regulation of *VEGFD* and *IL15* [48]. *IL6*, *CXCL8*, *SPP1*, *PRKAA* and *PRKCA* mRNAs were all reduced both in adherent cells and MCS grown in space. As VEGF-A was found increased and VEGF-D serum levels were reported to be reduced in patients suffering from high-stage thyroid cancer [93,94], the authors concluded that µ*g* possibly triggers some antitumor pathways involving IL-15 [48]. Consistent with these results a down-regulation of *VEGFA* was also found in the space-flown cells after the CellBox-2 mission, whereas the mRNA level of *VEGFA* stayed rather constant during a respective RPM experiment [54]. This VEGF discrepancy was often observed when results of simulated µ*g* experiments were compared with data from real µ*g* studies. Here it should be noted that VEGF gene expression is also up-regulated by the cells as a result of mechanical stress. It may be that fluid shear stress caused by random positioning [19,95,96] contributed to higher *VEGFA* mRNA levels of the RPM samples. In contrast to the space studies, the short-term exposure to real µ*g* during a parabolic flight suggested an increased malignancy of the FTC-133 cells [48].

### 4.4. Drug Targeting

Using proteome analysis, Pietsch et al. [97] were able to detect the expression of 37 proteins previously not found in thyroid cells. Of special interest is the plastin-2, which plays a crucial role in the development of thyroid tumours, as well as the interferon-induced 17 kDa protein precursor, which is coded by the *ISG15* gene [42]. This protein may be conjugated to target proteins by the E1 and E2 enzymes. Of the more than 150 yet known target proteins [98], we found 71 in the thyroid cells investigated [42]. During incubation on the RPM the protein’s accumulation decreased [45,56]. However, when mRNA expression was analyzed, after thyroid cells had been cultured for 10 days in space or on the RPM, a considerable up-regulation of the gene expression was observed [42,48,63]. ISG15 plays a role in antiviral immunity [99]. Recently a drug (Q63) was described, which exerted antiviral activity by down-regulation of ISG15 [100]. Even in cancer research ISG15 has attracted attention. It was recognized to be a prognostic marker in human breast cancer [101]. Studies on MCF7 cells suggested that the protein plays a role in stabilization of MCS generated on an RPM [65]. How the latter findings apply to thyroid cancer remains to be determined.

Bauer et al. [59] detected 69 proteins, which are significantly accumulated in thyroid cancer cells in the transition from 2D cell growth in normal gravity to spheroid growth in µ*g*. These factors seem to be part of an overarching mechanism and therefore they are very interesting candidates for targeted tumour therapy. Reduction or inhibition of metastasis by knock-down or knock-out of one of these factors would decisively improve cancer therapy as well as patient survival. After the exploratory phase researchers have now begun with the functional analysis of previously detected genes. Using pharmacological targeting or silencing of individual genes, they are able to inhibit selected signalling pathways. MCF-7 breast cancer cells which were incubated with the NFκB inhibitor dexamethasone showed dose-dependent inhibition of spheroid formation [37]. The artificial glucocorticoid dexamethasone functionally inhibits NFκB and NFκB-dependent gene expression. Based on these results in µ*g* research, it was proved that NFκB plays a crucial role in spheroid formation in breast cancer tumours. Further studies with the Src inhibitor PP2 and an anti-E-cadherin antibody also showed an influence on the spheroid formation of MCF-7 cells [65]. While the presence of PP2 completely inhibited spheroids, the E-cadherin antibody stimulated the formation of 3D structures. Both drugs had no effect on cell viability and all cells survived after pharmacological intervention. Similar attempts are planned for thyroid cancer research in the near future. The pharmacological substances that appear to be suitable are listed in Table 4.

## 5. Summary and Perspectives

Microgravity alters growth, cell adhesion, migration, proliferation, the cytoskeleton, extracellular matrix and focal adhesion of human cells in vitro. A large number of differentially regulated genes and altered protein synthesis and secretion are detectable in benign and malignant cells exposed to real or simulated µ*g*. The model system ‘Multicellular Spheroid’ is suitable to mimic metastasis of solid tumours and can be used for drug testing in pharmacology or as a coculture model to study processes like metastasis or angiogenesis. The investigation of the underlying mechanisms of spheroid formation also delivers valuable information about the in vivo cancer progression and metastasis.

Research in the fields of space medicine and gravitational biology makes it possible to find new proteins in organ tissues and changes in protein synthesis and secretion. Altered gravity conditions provide a new technology which is helpful to detect changes in proteins which may represent new targets for drug development in cancer. A large number of proteins which may serve as promising targets and available drugs are listed in Table 4. In order to address cancer growth and regulation, further µ*g* studies should be performed. We recently investigated changes of SOX transcription factors on FTC-133 follicular thyroid cancer cells exposed to the RPM and determined the mRNA expression of *SOX9* and *SOX11* in adherently growing and MCS cells [47]. The SOX family is involved in the progression and metastasis of cancer cells. Its expression in thyroid cancer is not clarified until today and requires further investigation. Studies focusing on the role of SOX family members and in particular the SoxF group in radioactive iodine-refractory differentiated thyroid cancer cells are of high interest. Recent results in the field of cancer research in µ*g* taught us that focal adhesions play an important role in the inhibition of proliferation and metastasis in melanoma cells [81]. Therefore, intensive research in thyroid cancer investigating focal adhesions should be performed in µ*g* by applying live-cell imaging with the FLUMIAS microscope [49] onboard the ISS or on sounding rockets.

Microgravity research is just one part of cancer research but might enable a significant step toward better treatments. Some years ago, with the help of simulated µ*g* various proteins were detected in thyroid tissue for the first time [97]. Some of them are targeted today. In addition, the compounds given in Table 4 are currently under investigation. The results may truly provide hope for thyroid cancer patients.

## Figures and Tables

**Table 1:** Selected articles addressing the mechanisms of spheroid formation of thyroid cancer cells exposed to real or simulated µ*g*.

Cell Line	Condition	Findings	Ref.
FTC-133	Space ISS (5d) preincubation (12d)	Factors involved in inhibition of 3D growth: caveolin-1, VCAM-1 and activated protein kinase Cα recruited in caveolae	[52]
FTC-133	Space ISS (5d) preincubation (12d)	Proteins involved in the inhibition of 3D growth: extracellular matrix proteins, phosphorylated profilin 1	[53]
FTC-133	Space (10d) Shenzhou 8	*IL6*, *CXCL8*, *IL15*, *SPP1*, *VEGFA*, *VEGFD*, *FGF17*, *MMP2*, *MMP3*, *TIMP1*, *PRKAA* and *PRKCA*	[48]
FTC-133	Space (10d) Shenzhou 8	*CTGF* and *EGF*	[63]
FTC-133	RPM 3d, (2d) preincubation (5d) preincubation	Vinculin, paxillin, focal adhesion kinase 1 and adenine diphosphate (ADP)-ribosylation factor 6	[45]
FTC-133, Nthy-ori 3-1	RPM (14d)	VEGF, FLT-1. FLK-1, CD44, Copine 1, TGM2, IL-6, IL-8, IL-17, OPN, neutrophil gelatinase-associated lipocalin (NGAL, LCN2)	[6]
ML-1, RO82-W-1	RPM (3d), FRC (7d)	ML-1 cells: elevated release of IL-6 and monocyte chemoattractant protein (MCP-1)	[64]

**Table 2:** Genes involved in spheroid formation.

Pathway/Function	Genes
Cell adhesion	*VCAM1*, *CD44*, *CDH1*
Angiogenesis	*VEGFD, VEGFA*, *FLK1*
Apoptosis	*TGFB1*
Caveolae	*CAV1*
Extracellular matrix	*SPP1*, *MMP2*, *MMP3*, *TIMP1*, *FN1*, *COL1A1*
Inflammation	*IL6*, *CXCL8*, *IL17*
NFκB signalling	*NFKB1*
Protein kinases	*PRKAA*, *PRKCA*
Cytoskeleton	*ACTB*, *TUBB*, *FN1*

**Table 3:** Comparison between exposure to µ*g* and metastasis.

	Microgravity (Detachment)	Metastases (Detachment)
Physical Trigger	Lapse of gravity (tensegrity, mechanical stress)	Pressure from growing tumour
Cytoskeleton	Formation of filopodia and lamellipodia [49]	Formation of filopodia and lamellipodia [85]
	*PFN*↑ [6], phosphorylated profilin-1 prevented MCS formation [43]	Profilin 1↓ [86]
Cell Adhesion	Blockage of E-cadherin leads to enhanced spheroid formation of MCF-7 breast cancer cells [65]	E-cadherin↓ [87]
ECM	*MMP9*↑ [43]; OPN↑ [61]	MMP9↑ [88]; OPN↑ [89]
Cytokines	IL-6↑, IL-8↑ [6]	IL-6↑ [87]; IL-8 enhances metastatic potential [90]
Growth Factors	VEGF-A↑ [48]	VEGF↑ facilitates metastasis through the VEGF-VEGFR1 signalling pathway [91]
Others	CD44↑ [61]	CD44↑ [92]

↓ down-regulation; ↑ up-regulation; AD: adherent cells; MCS: multicellular spheroids.

**Table 4:** Potential drugs targeting the proteins that were found to be influenced by µ*g*.

Drug	Target	Ref.
PP2 (4-amino-5-(4-chlorophenyl)-7-(dimethylethyl)pyrazolo[3,4-d]pyrimidine)	Proto-oncogene tyrosine-protein kinase Src	[102]
Daidzein	Caveolin-1	[103]
Camptothecin	Ubiquitin-like protein ISG15	[104]
SP600125	Mitogen-activated protein kinase 8	[105]
mNOX-E36	C-C motif chemokine 2	[106]
Dexamethasone, BAY 11-7082	NFκB p65	[107]
GSK2256098, MPAP	Focal adhesion kinase 1	[108]
MT189	Paxillin	[109]
Baicalein	Ezrin	[110]
Curcumin	HMOX-1	[111]
DX52-1	Radixin	[112]
TM5441	Plasminogen activator inhibitor 1	[113]
UK370106	Stromelysin, (MMP3)	[114]
Oseltamivir	Sialidase	[115]

## Abbreviations

2D	Two-dimensional
3D	Three-dimensional
ACTB	β-Actin
AD	Adherent cells
AMPK	5′Adenosine monophosphate-activated protein kinase
CAV1/2	Caveolin-1, -2,
CD44	Cluster of differentiation 44
CTGF	Connective tissue growth factor
CXCL	C-X-C motif
ECM	Extracellular matrix
EGF	Epidermal growth factor
ERK1/2	Extracellular signal-regulated kinase 1,2
FGF17	Fibroblast growth factor 17
FLK1	Fetal liver kinase 1
FLUMIAS	Fluorescence-microscopic analysis systems for space application
FLT1	Fms-related tyrosine kinase 1
FN1	Fibronectin
FRC	Fast-rotating clinostat
IL	Interleukin
KDR	Kinase insert domain receptor
MCS	Multicellular spheroid
MMP	Matrix metalloproteinase
µ*g*	Microgravity
Nek2	Serine/threonine-protein kinase Nek2
NFκB	Nuclear factor κ-light-chain-enhancer of activated B cells
OPN	Osteopontin
PRKAA	Protein kinase, AMP-activated, α catalytic subunit
PRKCA	Protein kinase Cα
RhoA	Ras homolog gene family, member A
RPM	Random positioning machine
RWV	Rotating wall vessel
SOX	Sex determining region Y-related high-mobility group box proteins
TGF-β	Transforming growth factor β
TIMP1	Tissue inhibitor of metalloproteinases 1
TLN1	Talin 1
TSH	Thyroid-stimulating hormone
TUBB	β-tubulin
VEGF	Vascular endothelial growth factor
